# Successful pulmonary valve regurgitation and pulmonary artery dilatation repair as a reintervention of pulmonary stenosis after 45 years

**DOI:** 10.1186/1749-8090-8-S1-P103

**Published:** 2013-09-11

**Authors:** I Duvan, S Ates, M Kurtoglu, BE Onuk, P Sungur, IS Karacan

**Affiliations:** 1 Department of Cardiovascular Surgery, Güven Hospital, Ankara, Turkey

## Background

Pulmonary Regurgitation (PR) accompanying with pulmonary artery dilatation is one of the most common complications after pulmonary valve stenosis repair during the late follow up period. PR is well tolerated first, but later it becomes the reason of the right ventricular dilatation and the morbid effects of it. Decision in the time of performing PVR is vitally important because severe PR ends up with RV volume overload and PVR is shown to be capable in reducing it and ameliorating the systolic activity afterwards. On the other hand PVR should have been performed as late as possible because of its limited time of life.

## Methods

A 57 year old man that underwent an operation of pulmonary comissurotomy for pulmonary stenosis and ASD closure 45 years ago admitted our clinic with the symptoms of congestive heart failure such as dyspnea, peripheral edema and oliguria. Hypertension, DM, hyperlipidemia, smoking and coronary artery disease were the other remarkable points of his medical past. He was hospitalised for a stent implantation of RCA in 2009 and because of atrial fibrillation due to congestive heart failure in 2011. Cardiac MRI findings were; severe PR, 48 mm PA, 101x76x60mm RV, 79x53x56mm LV and EF was 34.6%. Right and left ventriculography revealed that RVP and PAP were 35mmHg whereas PCWP was 24 mmHg. RCA had a lesion of 50% proximally and posterobasal akinesia in LV.

## Results

PVR (25 no Medtronic Bioprothesis Valve), Pulmonary Artery Aneurysmorrhaphy, CABGX1 and Tricuspid Kay Annuloplasty was performed. Aortic clamp time was 119, CPB time was 164 and the operation time was 330 minutes. Intubation period was 14, ICU stay was 46 hours, no (+) inotrope support was needed and he was discharged on the 6th day after.

## Conclusion

PVR and PA Aneurysmorrhaphy was performed successfully as a reintervention in this case for severe PR and PA dilatation, occurred in the late follow up period of pulmonary comissurotomy.

